# The Neuroprotective Effect of a Waste Byproduct Obtained From Pomegranate (*Punica granatum*)

**DOI:** 10.1002/ptr.70353

**Published:** 2026-05-12

**Authors:** Jessica Maiuolo, Valeria Mazza, Rosamaria Caminiti, Francesca Oppedisano, Saverio Nucera, Salvatore Ragusa, Sara Ilari, Lucia Carmela Passacatini, Luigi Tucci, Giuseppe Trunfio, Vincenzo Mollace, Carolina Muscoli

**Affiliations:** ^1^ Department of Health Science, Institute of Research for Food Safety & Health (IRC‐FSH) University Magna Graecia of Catanzar Catanzaro Italy; ^2^ PLANTA/Research Documentation and Training Center Palermo Italy; ^3^ Department of Human Sciences and Quality of Life Promotion San Raffaele University Rome Italy; ^4^ Pain Physiology and Pharmacology IRCCS San Raffaele Roma Rome Italy

**Keywords:** circular economy, gut microbiota, neurodegeneration, neurodegenerative diseases, polyphenols, pomegranate peel

## Abstract

Pomegranate is an exceptional fruit that can have several beneficial effects on human health. The peel of pomegranate, a waste product, should be recovered as it still contains valuable constituents, including phenolic compounds, minerals and fibre. The recovery of bioactive compounds occurs through eco‐sustainable extraction techniques that reduce costs and promote environmental sustainability and public health. Neurodegeneration is a pathological process causing progressive neuronal damage, potentially leading to cell death. Currently, no drug is known to cure neurodegenerative diseases definitively. A balanced diet rich in fruits and vegetables is associated with a lower risk of certain neurodegenerative conditions. The phytochemicals of plants and their beneficial bioactive compounds demonstrate promising therapeutic potential for human neurological diseases, exhibiting antioxidant and anti‐inflammatory properties in the brain. This review aims to highlight the currently existing knowledge on the effects of pomegranate peel against neurodegenerative diseases and their mechanisms of action. The manuscript is intended for those who explore strategies for recovering waste materials and for those who investigate the effects of natural products on neurodegeneration. Prospects could include clinical trials in which pomegranate peel is used in the management of human neurodegenerative diseases.

## Graphical abstract

The Effect of a Waste Byproduct Obtained From Pomegranate on Neurodegeneration.

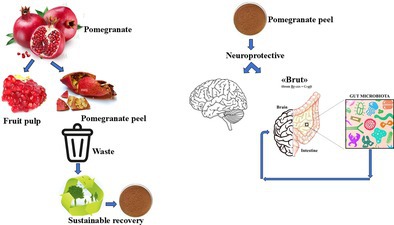

## 1 Introduction

The pomegranate (*Punica granatum*, L.) is a member of the *Lythraceae* family (formerly *Punicaceae*) (Ge et al. 2021), belonging to the genus Punica. It originated in the Himalayas of Northern India; since then, it has been widely cultivated across Central Asian countries and throughout the Mediterranean region (Faria and Calhau 2011). In various cultures, the pomegranate symbolises themes like life, immortality, well‐being, femininity and fertility (Karimi et al. 2017). Originally, pomegranate was used as an anti‐parasitic, anti‐helminthic and vermifuge (Eddebbagh et al. 2016), to treat ulcers, dysentery and diarrhoea (Abid et al. 2018). As knowledge about pomegranate spread, people began to use it for a range of health issues, including oral diseases, peptic ulcers, diabetes, hyperlipidemia, hypertension and certain types of cancer, thanks to its anti‐inflammatory, antioxidant, antibacterial and antiviral properties (Moga et al. 2021).

The benefits of pomegranate stem from its diverse components, including polyphenols, amino acids, minerals, vitamins, fibre, flavonoids, unsaturated fatty acids, anthocyanins, alkaloids, steroids, phenylpropanoids, terpenes, organic acids, sterols and others (Sharma and Maity 2010). However, it should be emphasised that the exact composition of the pomegranate depends on several factors, including cultivar, ripening stage, climate, cultivation techniques, soil characteristics, processing and storage conditions (Kandylis and Kokkinomagoulos 2020). The rich composition of pomegranate is the basis of many beneficial activities that improve human health, including antioxidant, anti‐inflammatory, anticancer, antiviral and antibacterial properties (Šavikin et al. 2018). Furthermore, more recently, the protective effects of pomegranate against metabolic diseases (diabetes, hypertension and hyperlipidemia), cardiovascular disorders and neurodegeneration have also been positively evaluated (Cordiano et al. 2024). Pomegranates are considered functional foods, with edible portions (50%) and juice yields between 36% and 63%, depending on location and season (Khwairakpam et al. 2018). The edible part of the pomegranate fruit is made up of 40% internal grains (aryllis) and 10% seeds. The other 50% of the total weight of the fruit corresponds to the inedible portion, including the peel, the hardest external part and the internal white part, which surrounds the grains (Melgarejo et al. 2021). The peel is rich in nutrients, including phenolic compounds (flavonoids, ellagitannins and proanthocyanidins), minerals (such as potassium, calcium, phosphorus, sodium and magnesium) and fibre (Man et al. 2022). The plant kingdom is the primary producer of waste, including parts of the plant that are not utilised, such as leaves, peels, deteriorated roots, membranes that separate portions of the fruit, processing residues and seeds (Tomar et al. 2023). In reality, what is commonly considered plant ‘waste’ could be recovered and would contribute to a more sustainable economy: plant waste is rich in substances and can be reused to produce compost, making the soil more fertile, biopesticides, biogas for thermal and electrical energy, biomethane for transport and packaging materials (Castro‐Muñoz et al. 2022). Finally, waste can also provide agri‐food by‐products, responsible for the synthesis of nutraceuticals, supplements, functional foods and cosmetics (Fraga‐Corral et al. 2021; Zhou et al. 2023). The recovery of bioactive compounds occurs through eco‐sustainable extraction techniques that reduce costs and promote environmental sustainability and public health. These bioactive compound recovery mechanisms reflect the circular economy production model, which aims to extend the life cycle of products and resources while minimising waste generation through the reuse, reconditioning and recycling of existing materials (Tanveer et al. 2022).

Neurodegeneration is a pathological process causing progressive neuronal damage, potentially leading to cell death. Neurodegenerative diseases share a common feature: neurodegeneration, leading to functional, cognitive and motor impairments (Querfurth and Lee 2021). There are many causes responsible for neurodegeneration; among these, it is worth mentioning:

- Buildup of abnormal proteins: in some pathologies, such as Alzheimer's disease, the misfolded proteins accumulate and damage neurons (Hetz and Saxena 2017).
- Unstable molecules, called free radicals, have short lifespans and cause damage (MohanKumar et al. 2023).
- Neuroinflammation, a chronic brain inflammatory process involving the immune system, may cause neurodegeneration and poses a serious risk (Patani et al. 2023).
- Mitochondrial dysfunction, from morphological or functional damage, compromises their activity and can lead to neurodegeneration (Klemmensen et al. 2024).
- Acute and chronic brain injury resulting from a traumatic insult (Brett et al. 2022).
- Lysosomal system alteration (autophagy) (Somogyi et al. 2023).
- Gene mutations causing neuronal dysfunction underlie some neurodegenerative diseases (Verheijen et al. 2018).

**FIGURE 1:**
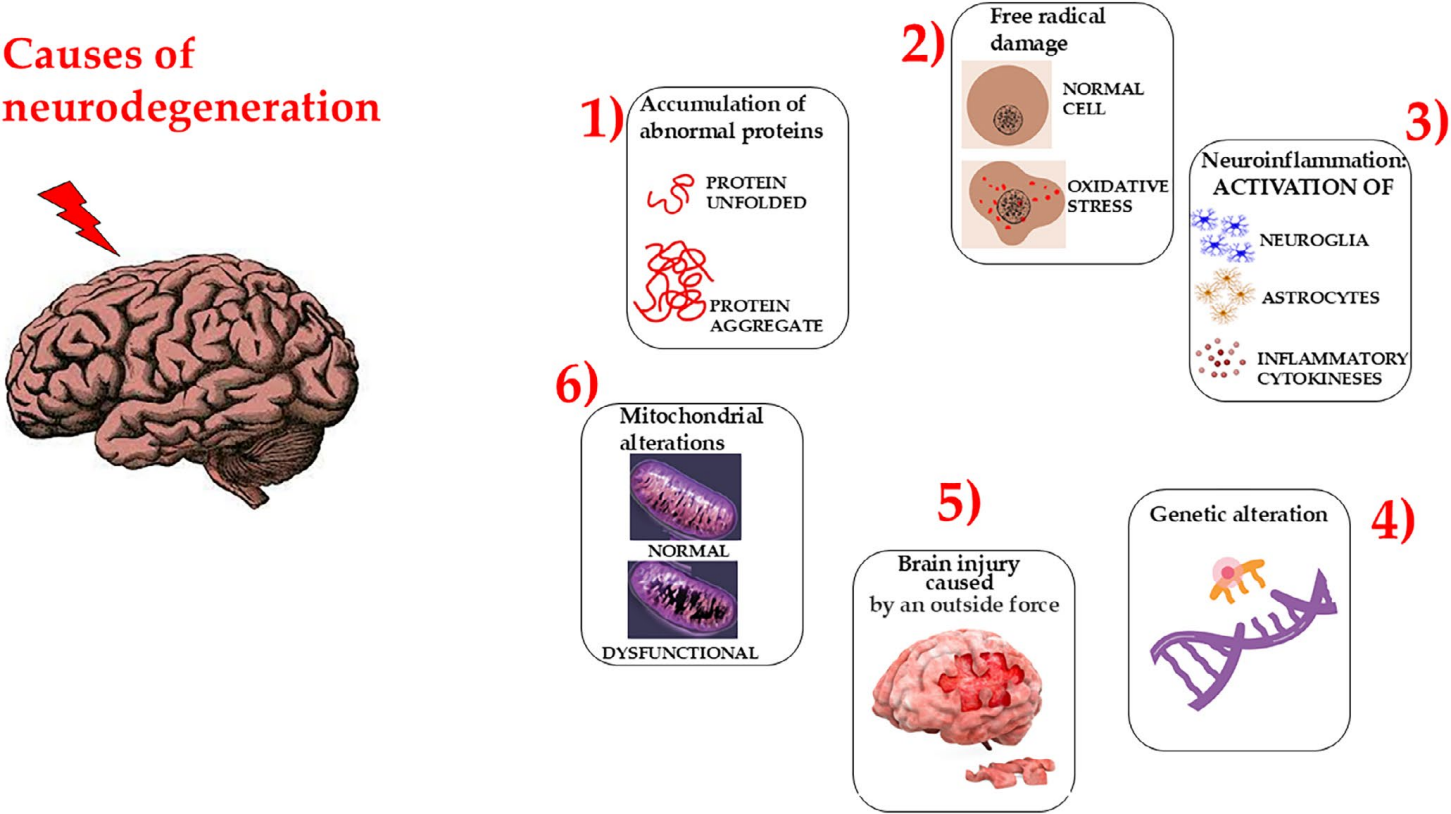
Neurodegeneration. The illustration depicts the primary causes of neurodegeneration impacting the brain.

Currently, no drug is known to cure neurodegenerative diseases definitively. Some lifestyles may help reduce your chances of developing these diseases (Breijyeh and Karaman 2020); for example, a balanced diet is associated with a lower risk of some neurodegenerative conditions. A diet focusing on fruits, vegetables, monounsaturated fats, fish, whole grains, legumes, nuts, moderate alcohol intake and limited red meat, dairy, saturated fats and refined cereals may help protect the brain (Gardener and Caunca 2018). These foods are characteristic of a Mediterranean diet and may help combat neurodegenerative diseases (Więckowska‐Gacek et al. 2021). Phytochemicals, the bioactive compounds found in plants, exhibit promising therapeutic potential for various human diseases by potentially preventing or treating different pathological aspects (Fierascu et al. 2020). These substances often exhibit antioxidant and anti‐inflammatory effects in the brain, activating protective mechanisms (Limanaqi et al. 2020; Abdolmaleky and Zhou 2023). Plants contain approximately 10,000 polyphenol structures, natural compounds characterised by at least one phenolic group (Silva and Pogaˇcnik 2020; Dretcanu et al. 2021). Plants produce polyphenols as secondary metabolites featuring a single phenolic group (C_6_H_5_OH), where a hydroxyl replaces a hydrogen on a benzene ring (Mahajan et al. 2018). Polyphenols provide numerous health benefits, including antioxidant, anti‐inflammatory, antimicrobial, antidiabetic and anticancer effects (Tatipamula and Kukavica 2021; Fraga et al. 2018). Additionally, polyphenols can reduce cardiovascular risk and influence the regulation of lipids and glucose (Iqbal et al. 2023; Ma et al. 2023). However, the effectiveness of these benefits largely depends on what happens to the polyphenols after ingestion and how well the body can absorb them (Lippolis et al. 2023; Williamson 2025). A summarised diagram of the neurodegeneration process is shown in Figure 1.

This review aims to highlight the currently existing knowledge on the effects of pomegranate peel against neurodegenerative diseases. Can pomegranate peel considerably slow the progression of neurodegeneration? In this case, what are the mechanisms involved? In this review, we aim to address these questions. The manuscript is aimed not only at those who delve into strategies for recovering waste materials, but also at those who study the effects of natural products on neurodegeneration.

## 2 General Characteristics of the Pomegranate

### 2.1 Bioactive Compounds

Bioactive compounds in natural foods regulate cellular metabolic processes, bringing beneficial effects to human health. Pomegranate contains many phytochemicals, and this characteristic accentuates some protective effects; for this reason, the fruit is identified as a ‘superfruit’ (Wei et al. 2024). Globally, polyphenols can interact with each other or with other bioactive components, producing a combined interaction that yields a total synergistic effect greater than the sum of the effects of individual molecules (Song et al. 2022; Radwan et al. 2025). The numerous bioactive compounds of pomegranate can be categorised into two primary groups: phenolic compounds and fatty acids. Phenolic compounds are a large class of organic molecules of plant origin, structurally characterised by an aromatic ring with one or more hydroxyl groups. Polyphenols perform crucial functions for their well‐being (acting on colour, defence, support) and offer important antioxidant benefits for human health. Two subgroups of phenolic compounds are hydrolyzable tannins and flavonoids. Hydrolyzable tannins are polyphenolic substances which are derived from gallic acid (3, 4, 5‐trihydroxybenzoic acid) and, when subjected to hydrolysis, split into sugars and phenolic acids such as gallic acid or ellagic acid, included in gallotannins and ellagitannins, respectively; gallotannins can be hydrolysed into gallic acid, known for its powerful antioxidant, anti‐inflammatory and antimicrobial properties (Piazza et al. 2022). Ellagitannins neutralise free radicals, possess protective properties for cellular mitochondria, are implicated in the resolution of certain metabolic diseases, and are associated with anti‐inflammatory, cardioprotective and chemopreventive effects (Kiss and Piwowarski 2018). The most abundant type of ellagitannin is punicalagin, which can be hydrolysed into smaller phenolic compounds such as ellagic acid. Punicalagin is a natural bioactive of great interest for health and cosmetics, thanks to its high biological activity, which translates into antioxidant, anti‐inflammatory, antitumor and antimicrobial effects (Xu et al. 2021; Alalawi et al. 2023). Punicalagin can be hydrolysed into smaller phenolic compounds, the most famous of which is ellagic acid. Ellagic acid is present in every portion of the pomegranate. It is responsible for the increased number of bioactivities, exhibiting positive effects against different types of cancer (Yin et al. 2024; Čižmáriková et al. 2023), possessing antiallergic, antidiabetic, anti‐inflammatory, antimicrobial and anti‐tyrosinase properties and has also been linked to protection against neurodegenerative diseases (Xue et al. 2022). Data on the absorption of ellagic acid are conflicting due to its almost zero solubility in the gastric environment and poor solubility in the intestinal environment. However, thanks to intestinal bacteria, ellagic acid is transformed into urolithins (A, B, C and D) (Tomás‐Barberán et al. 2017), molecules with powerful beneficial effects, especially related to mitophagy and anti‐ageing (Tow et al. 2022). Urolithins result from the transformation of ellagitannins through lactone‐ring cleavage, decarboxylation and dehydroxylation reactions by intestinal microbiota (García‐Villalba et al. 2023). Flavonoids are a large group of secondary plant metabolites, antioxidant and anti‐inflammatory pigments belonging to the polyphenol family, responsible for bright colours (from yellow to purple) and offering benefits for cardiovascular, brain and cellular health. They protect against free radicals and reduce the risk of chronic diseases. The main flavonoids of pomegranate are anthocyanins (cyanidin and delphinidin), luteolin, quercetin, rutin, kaempferol, catechin and epicatechin (Shen et al. 2022). Anthocyanins are water‐soluble natural pigments belonging to the flavonoid family. Chemically, they are glycosides, sugars linked to a non‐sugar portion called anthocyanidin, and change colour based on pH (Naróg and Sobkowiak 2023). They are responsible for the red colours of pomegranate fruit and its juice, although the intensity of the colour depends on the type of anthocyanin (e.g., Delphinidin derivatives are responsible for the blue and purple colour, while Pelargonidin is related to the red‐orange colour), and its concentration (Wu et al. 2024). Anthocyanins act as powerful antioxidants, protecting cells from damage caused by free radicals. The antioxidant activity of these compounds is three times greater than that of red wine, and overall, together with the other phytochemicals contained in pomegranate, they guarantee an extremely high total antioxidant capacity (Wang, Wang, et al. 2024). Seeram et al., found that, considering some foods with antioxidant activity, it was possible to construct the following order with the greatest antioxidant effect towards the least: pomegranate > red wine > grape juice > blueberry juice > cranberry juice > orange juice > apple juice (Seeram et al. 2008). Luteolin is one of the most common flavonoids and is characterised by a double bond between C2 and C3. Luteolin of pomegranate has been shown to have antioxidant, antimicrobial, anti‐inflammatory, anti‐neurodegenerative and antitumor activities, suppressing uncontrolled cell growth, inhibiting cell migration and reducing metastasis formation (Jayawickreme et al. 2024; Wang, Li, et al. 2024). The absorption of luteolin is very rapid, while its elimination is slow, demonstrating a long‐lasting permanence after ingestion (Zhu et al. 2024). Finally, it was shown that luteolin effects were higher when the entire pomegranate fruit was consumed, compared to taking the pure compound in an industrially produced supplement (Hayasaka et al. 2018).

The second main group of pomegranate bioactive compounds consists of fatty acids. Pomegranate fatty acids are predominantly polyunsaturated and play a crucial role in preventing various human diseases. The most important compound, contained in pomegranate, is punicic acid. Punicic acid is an isomer of alpha‐linolenic acid, predominantly abundant (up to 80%) in pomegranate seed oil. Chemically, it consists of three double bonds (in the *cis*‐9, *trans*‐11 and *cis*‐13 positions) and exhibits remarkable antioxidant, anti‐inflammatory, antitumor, antidiabetic and skin revitalising properties (Holic et al. 2018). The absorption of this compound is quite slow, and its bioactivity appears to be correlated with its conversion to conjugated linoleic acid (Pereira de Melo et al. 2019).

### 2.2 Beneficial Properties of Pomegranate

The bioactive compounds contained in pomegranate can exert numerous health benefits, as demonstrated by clinical and preclinical studies; the main ones are described below:

#### 2.2.1 The Antioxidant Activity

The antioxidant property of pomegranate is achieved thanks to its ability to reduce reactive oxygen species. This activity occurs following the involvement of numerous pathways, including the one that causes upregulation of the NAD‐dependent deacetylase protein sirtuin‐3, also known as SIRT3, a member of the sirtuin family (Velpuri et al. 2024). The human sirtuins have a range of molecular functions and have emerged as important proteins in ageing, stress resistance and metabolic regulation. SIRT3 is a soluble protein located in the mitochondrial matrix, and its overexpression increases respiration and also decreases the production of reactive oxygen species (Shen et al. 2020). SIRT3 is responsible for activating antioxidant enzymes such as superoxide dismutase 2 (SOD 2) (Zhao et al. 2016). When cellular production of ROS overwhelms the antioxidant capacity, this leads to a state of oxidative stress, and the cell activates some proteins, including the transcription factor nuclear factor‐κB (NF‐κB). Pomegranate intake, on the other hand, is responsible for the inhibition of NF‐κB with a clear projection towards the antioxidant direction (Feng et al. 2020; Chen et al. 2024). Some clinical studies have highlighted that the consumption of pomegranate and its bioactive compounds has reduced lipid peroxidation metabolites (such as malondialdehyde, MDA), increased both the total antioxidant capacity (TAC, the overall effectiveness of a sample in neutralising free radicals) and the activity of the enzyme paraoxonase‐1 (PON1, whose activity is the measurement of the function of this enzyme linked to HDL, crucial for antioxidant defense) (Sohrab et al. 2017). Another pathway that leads to the antioxidant effect of pomegranate is the activation of the Peroxisome Proliferator‐Activated Receptor gamma (PPARγ) following the ingestion of this fruit or its juice. PPARγ is a nuclear receptor and key transcription factor that regulates overall cellular health, metabolism, cellular differentiation and inflammation, acting as a master switch for the expression of genes involved in maintaining cellular homeostasis (Li et al. 2020).

The antioxidant activity of pomegranate is also exploited in the food sector, and this fruit is used as an additive, exploiting it to increase the shelf life of some foods, such as meat, bread and ice cream (Kanatt et al. 2010; Sagdic et al. 2012).

#### 2.2.2 The Anti‐Inflammatory Activity

Numerous studies have shown that pomegranate extracts were able to prevent or reduce the deleterious effects of some types of inflammatory diseases. For example, in vitro studies have shown that inflammation triggered by lipopolysaccharide (LPS), an outer membrane molecule of Gram‐negative bacteria and a potent stimulator of the immune system, was reduced by treatment with a pomegranate extract. The latter reduced inflammatory markers, such as nitric oxide (NO), Prostaglandin E2 (PGE2), Interleukin‐1 beta (IL‐1β), IL‐6 and Tumour necrosis factor alpha (TNF‐alpha) (Xu et al. 2017). These results were confirmed by in vivo studies performed on rats in which colitis was pharmacologically induced. After treatment with a pomegranate extract, animals showed preventive effects on disease development, reduction of biochemical markers, including NO levels, neutrophil infiltration, gene expression of IL‐1β, IL‐18, TNF‐α and NF‐κB (Marín et al. 2013). Interestingly, these effects were achieved both following the administration of pomegranate juice (400 mg/kg) and with purified punicalagin (4 mg/kg, the amount contained in pomegranate juice). However, the protective effect of pomegranate juice was greater than that of punicalagin alone, demonstrating that several phytochemical components contributed synergistically to the desired effect (Shah et al. 2016). Ellagic acid (100 mg per day for 7 days) demonstrated similar protective effects on a mouse model of ulcerative colitis, significantly reducing intestinal inflammation markers, such as Cyclo‐Oxygenase 2 (COX‐2), inducible nitric oxide synthase (iNOS) and other pro‐inflammatory markers expression (Jeong et al. 2023). In addition to ellagic acid, other secondary metabolites, such as urolithin A, punicalin, grantin B, strictinin and punicalagin, may contribute to the observed anti‐inflammatory effect (Li, Ruan, et al. 2023). Clinical trials of ulcerative colitis patients also showed a symptomatic reduction (faecal incontinence, diarrhoea and rectal bleeding) after treatment with pomegranate extract (6 g/day for 4 weeks) (Larrosa et al. 2010).

#### 2.2.3 Neuroprotective Effects

Scientific literature has highlighted the role of pomegranate in neurodegenerative protection. Specifically, the bioactive components of this fruit, particularly ellagic acid, can regulate neurotransmitter metabolism. An in vitro analysis highlighted how ellagic acid, obtained from various pomegranate extracts, exhibits an inhibitory function against cholinesterase (Kamali et al. 2015). An interesting in vivo paper, conducted on obese rats due to a high‐fat and high‐fructose diet, confirmed the protective effects of pomegranate extracts, capable of inhibiting the brain cholinesterase enzyme and increasing antioxidant capacity (Konsoula 2018). Similarly, ellagic acid has demonstrated protective actions in both Alzheimer's disease and Parkinson's disease (Amri et al. 2017; Jha et al. 2018). Ellagic acid administration was also protective in experimental autoimmune encephalitis, the most common model for multiple sclerosis, delaying disease progression and reducing inflammatory cell infiltration into the central nervous system (Baluchnejadmojarad et al. 2017).

#### 2.2.4 Effects on the Cardiovascular System

The effects of pomegranate extracts or their bioactive compounds on the cardiovascular system have been extensively studied. Overall, pomegranate extracts containing at least 30% punicalagin were shown to significantly reduce endothelial dysfunction caused by a high‐fat diet (Busto et al. 2018). In parallel, a pomegranate extract (500 mg/kg, administered for 6 weeks) to overweight and diabetic rats reduced triglycerides and fatty acids, thereby improving altered cardiac lipid metabolism (Vilahur et al. 2015). Treatment of rats with isoproterenol, a drug that enhances the action of the heart, increased heart rate and induced arrhythmias, when also supplemented with pomegranate juice (20 mL/day, for 1 month before receiving the drug), demonstrate less tissue necrosis, an increase in reduced glutathione, vitamin C, Superoxide Dismutase and catalase enzymes: all these results suggested a cardioprotective action of pomegranate juice (Huang et al. 2005). Pomegranate juice also protected rats exposed to cigarette smoke (for 1 month), showing less cardiac hypertrophy, aortic calcification than the control and overall an anti‐atherosclerotic action (Jadeja et al. 2010). Clinical studies have confirmed the effects described in in vivo experiments. For example, a study was conducted in which 100 adults with myocardial infarction were supplemented for 5 days with 220 mL/day of pomegranate juice, containing 840 mg/g of dry weight of polyphenols and particularly rich in ellagic acid (about 88.8%). Pomegranate juice intake ensured an improvement in cardiac damage, a reduction in the incidence and severity of angina pectoris episodes, and a reduction in some blood markers (TNFα, IL‐6, MDA, troponin) (Al Hariri et al. 2016).

Cardiometabolic risk factors include several conditions, such as dyslipidemia, obesity, hypertension, hyperglycemia and insulin resistance. These alterations increase the risk of developing numerous cardiovascular diseases, type 2 diabetes and metabolic syndrome (Razani et al. 2017; Virgen‐Carrillo and Mojica 2025). Hyperglycemia is a metabolic condition highly related to diabetes mellitus in which glucose regulation is altered due to increased hepatic glucose production, impaired glucose uptake, reduced insulin secretion, or reduced glucose utilisation in peripheral tissues. Pomegranate intake has demonstrated hypoglycemic potential by improving insulin production, glucose absorption and utilisation (Hamo et al. 2024). In an in vivo model of rats with type 2 diabetes and fed a high‐fat diet, administration of pomegranate, or its bioactive compounds punicalagin, ellagic acid and urolithin A, increased insulin secretion (Mouri and Badireddy 2024). Exposure to flavonoids, tannins and organic acids reduced glucose uptake and glucogenesis (Zhang, Hou, et al. 2021). Insulin sensitivity was also improved by arils juice (7.8 mg punicalagin/kg/day for 6 weeks (Bandawane et al. 2023), administered to diabetic rats). Furthermore, pomegranate reduced pancreatic β‐cell apoptosis, improving insulin production. In diabetic mice, treatment with 50 mg/kg/day of urolithin A for 8 weeks reduced inflammation and β‐cell death (El‐Beih et al. 2019). Pomegranate affects glucose levels, increasing glycolysis and decreasing gluconeogenesis (Zhang et al. 2021). Finally, another interesting study (Zhang et al. 2020) involved the administration of pomegranate juice (0.15% in drinking water for 4 weeks) to obese rats, achieving a reduction in glucose transport and hepatic gluconeogenesis.

Hyperlipidemia is a metabolic disorder characterised by high levels of lipids in the blood, which increases the risk of cardiovascular disease.

Pomegranate has lipid‐lowering potential, reducing cholesterol and triglyceride levels. Pomegranate seed oil is rich in punicic acid, and its administration to hyperlipidemic mice resulted in a reduction in the synthesis of triacylglycerol and cholesterol (Ahmed et al. 2015). In an in vitro study conducted on the human hepatocyte cell line, a strong lipid‐lowering effect from ellagic acid through the increase in cholesterol metabolism (Franczyk‐Żarów et al. 2023). Additionally, a pomegranate extract, enriched with 40% punicalagin and administered to male rats fed a fat diet, reduced the concentration of hepatic cholesterol and triglycerides (Lv et al. 2016). Endothelial dysfunction is a feature of early atherosclerosis and was prevented by a pomegranate extract (200 mg punicalagin/day), administered for 10 days to pigs fed a hypercholesterolemic diet (Zou et al. 2014). Another mechanism for reducing cholesterol accumulation is increased cholesterol efflux from cells. Zhao et al. demonstrated that punicalagin (20 and 40 μg/mL) was able to increase cholesterol efflux into steatotic liver cells (Zhao et al. 2014). The same result was obtained in in vivo experiments on hamsters fed a hyperlipidemic diet; cholesterol efflux was induced after administration of ellagic acid (44, 88 and 177 mg/kg/pc) (Zhao et al. 2014).

Obesity is characterised by an abnormal accumulation of body fat or adipose tissue, which increases the risk of noncommunicable diseases, including cardiovascular pathologies (Liu et al. 2015). The pomegranate has anti‐obesity potential, which is attributed to its ability to reduce lipid synthesis. Punicalagin (10, 20 μM and 4.2 IC50 μg/mL), ellagic acid (10, 50 μM and 1.31 IC50 μg/mL), and urolithin A (10, 50 μM) have reduced adipogenesis in a preadipocyte model in a dose‐dependent manner and intracellular triglycerides (Chamarthi and Daley 2025). Punicalagin downregulated lipid synthesis and activated lipolysis, resulting in a decrease in hepatic lipid deposition in obese mice after 8 weeks of exposure (Li et al. 2020). A recent therapeutic approach to reduce obesity has considered increasing mitochondrial function in adipose tissue as a mechanism to increase energy expenditure; with this aim, obese mice were treated with urolithin 30 mg/kg/day for 10 weeks (Jiensi et al. 2023).

#### 2.2.5 Anticancer Effects

Pomegranate inhibits the growth of several types of tumour cells, including prostate, breast, pancreatic, lung, colon and hepatocellular cells, through several mechanisms (Echeverria et al. 2021; Teniente et al. 2023). For example, the antitumor properties of pomegranate against breast cancer have been attributed to anti‐aromatase and antiestrogenic activity, since it is well known that high estrogen levels represent one of the main risk factors for the growth of this tumour (Pantiora et al. 2023).

In recent years, polyphenols of pomegranate have been tested for their antiproliferative effects and among the tannins, the most potent enzyme inhibitor was urolithin B, followed by gallagic acid (Adams et al. 2010). Jeune et al. studied the antitumour activity of pomegranate extract, in combination with the isoflavone genistein, in MCF‐7 breast cancer cells (Jeune et al. 2005). After 24 h of treatment with pomegranate juice and genistein, in single and combined treatments, the cytotoxic effect and growth reduction were quantified: the results revealed that pomegranate extracts, plus Genistein was able to induce cell apoptosis (Eroglu Ozkan et al. 2021).

Punicalagin, ellagic acid and total pomegranate tannins also showed apoptosis and antiproliferative actions on human colon, oral and prostate cancer cells (Jeune et al. 2005). Dahlawi et al. demonstrated that pomegranate extract induced apoptosis in leukaemic cell lines (Dahlawi et al. 2012; Seeram et al. 2005), while Li et al. evaluated that pomegranate leaf extract induced apoptosis in lung cancer cells, together with cell cycle arrest in the G2/M phase (Li et al. 2016).

Antioxidant and anti‐inflammatory effects have contributed to the antitumour effect. Several studies have found that COX2 overexpression, closely associated with cancer metastasis, could be significantly attenuated by treatment with pomegranate extract, rich in polyphenols (107.5 ± 3 mg/g tannic acid equivalents) and had antioxidant activity (Li et al. 2016; Shukla et al. 2008). Furthermore, the anti‐inflammatory effect of pomegranate was able to counteract metastases of colon or prostate cancer (Hajleh and Al‐Dujaili 2016). Ellagitannins were found in high amounts in prostate tissue, suggesting that pomegranate metabolites may play an antitumor role in this tissue. Following pomegranate juice ingestion, urolithins A and B were concentrated in the prostate, accentuating their beneficial role against prostate cancer (Thotambailu et al. 2019). The most common side effects of radiotherapy are mucositis and dermatitis. One study found that patients treated with 2 capsules/day of 300 mg pomegranate extract (containing 40% polyphenols and 27% punicalagin) for 6–7 weeks exhibited a protective effect in preventing radiation therapy‐induced skin damage. In general, in the presence of neoplasms, consumption of pomegranate fruit determines an activation of the proinflammatory transcription factor NF‐kB and an overexpression of caspase‐3. It increases the blockade of the G0/G1 phase of the cell cycle (Pacheco‐Palencia et al. 2008).

## 3 Pomegranate Peel: A Precious Resource

A significant part of fruit and vegetables is discarded and considered as waste (Chen et al. 2022; Teplitski et al. 2023). Since these wastes can be recovered and reused for various purposes, a currently prevalent challenge is identifying potentially protective molecules present in by‐products and wastes from the agri‐food industry (Maiuolo et al. 2023). Therefore, pomegranate peels are considered agro‐industrial waste, adding to environmental pollution from improper disposal. Nevertheless, the biological activity of pomegranate peel is even greater than that of pomegranate pulp, due to its chemical composition, which is particularly rich in secondary metabolites (Angeloni et al. 2022). For example, literature data have highlighted an antioxidant activity of pomegranate peel almost 10 times higher than that of the other parts of the pomegranate fruit and this property is closely linked to its chemical and polyphenolic structure (Azmat et al. 2024). The most common compound families found in pomegranate peel are: tannins, steroids, polyphenols, flavonoids, dietary fibre, anthocyanins, phenolic acids, alkaloids, terpenoids, catechins, vitamins, minerals and saponins (Hanafy et al. 2021), while the most represented individual phytochemicals are: ellagic acid, punicalins, punicalagins, gallic acid, p‐coumaric acid, syringic acid, shikimic acid, chlorogenic acid, butyric acid, benzoic acid, caffeic acid, cinnamic acid, protocatechuic acid, ferulic acid, quinic acid, rutin, resorcinol, quercetin, vitamin A, vitamin B1, vitamin B2, vitamin C, vitamin E, calcium, potassium, sodium, phosphorus, iron (Mo et al. 2022; Singh et al. 2023; Senobari et al. 2022; Tomás‐Barberán et al. 2017; Cortés‐Martín et al. 2020; Palikaras et al. 2018; Magangana et al. 2020). The fibre, a significant component with established health benefits, is also found in various plant byproducts (Kujawska and Jodynis‐Liebert 2020; Wang, Long, et al. 2024); its content in pomegranate peel varies from 33% to 62% (Caminiti et al. 2024), although its concentrations depend on the cultivar, ripening stage, climate, soil composition and nutrient analysis methods (Maiuolo, Liuzzi, et al. 2024; Xiong et al. 2023). Appropriate analyses have established that this fibre is composed primarily of lignin, cellulose, uronic acid and neutral sugars. It has been calculated that the ratio between the insoluble and soluble fibre is very close to 1 (Al‐Rawahi et al. 2013). The role of fibre in pomegranate peel is mainly to absorb cholesterol, actively participating in the protective role of the cardiovascular system (Maiuolo et al. 2024). The pomegranate peel, as already mentioned, has several protective effects, including antioxidant, anti‐inflammatory, anti‐diabetic, hypo‐lipidic, neuroprotective, cardioprotective, anti‐apoptotic, anti‐mutagenic, anti‐genotoxic, anti‐cancer, anti‐viral and anti‐bacterial properties, potentially mitigating various chronic illnesses (Hasnaoui et al. 2014; Neyrinck et al. 2019). These countless beneficial properties, provided by the pomegranate peel, make this portion of the fruit a precious and irreplaceable part. The anti‐oxidant and anti‐inflammatory activities are particularly interesting since oxidative processes and the onset of inflammation are the common denominator of most pathologies. In particular, these two elements underlie the onset, development and progression of neurodegeneration (El‐Hadary and Ramadan 2019). The results in the literature allow us to consider the bioactive compounds of pomegranate peel as components that oppose oxidative stress; in fact, the valuable constituents reduce/or eliminate free radicals, prevent the oxidation of biological macromolecules, transform toxic free radicals into less toxic compounds and increase the expression of antioxidant enzymes (Bido et al. 2021; Marogianni et al. 2020; Sun et al. 2017).

Ellagitannins are poorly bioavailable; they have a high molecular weight (300–3000 g/mol) and are poorly absorbed in the upper gastrointestinal tract. In the stomach and small intestine, they are partially hydrolysed into ellagic acid, the absorption of which is, however, limited. Most ellagitannins reach the colon intact, where the bacterial flora transforms them into urolithins. The latter are much more bioavailable and are primarily responsible for systemic beneficial effects (Elwej et al. 2016). Ellagitannin metabolites are eliminated mainly by the renal and faecal routes, but some compounds may persist in urine after ingestion; these metabolites show low toxicity and minimal side effects (Saparbekova et al. 2023). In contrast, anthocyanins have a low molecular weight (< 500 Da) and good potential inhibition of inflammatory and neurodegenerative pathways. They are absorbed in the intestine and are distributed in various tissues, showing beneficial effects on the heart, brain and eyes. They are rapidly metabolised in the liver and intestine into compounds such as phenolic acids, which retain some of their biological activity. They are excreted mainly through urine and bile and have low toxicity and mutagenicity (Aguilar‐Zarate et al. 2018; Roberts et al. 2020). Finally, concerning fatty acids, it can be stated that the short and medium chain molecules are absorbed directly into the portal blood, while the long chain ones require the formation of micelles with bile salts to enter enterocytes. They can be stored as triglycerides in adipose tissue or incorporated into plasma membranes as phospholipids. Fatty acid metabolism preferably occurs through beta‐oxidation in mitochondria, which converts fatty acids into acetyl‐CoA for energy production. Small amounts of water‐soluble metabolites can be excreted renally. High levels of free fatty acids can cause lipotoxicity, contributing to insulin resistance and metabolic diseases (Kumkum et al. 2024; Schönfeld and Wojtczak 2016).

Pomegranate peel has been used not only in the production of dietary products, supplements, edible coatings, flavours, colours and films for food packaging (Bremer and Norum 1982), but also as innovative products for making silver nanoparticles, for nanotechnology, bioengineering, catalysis and electrochemistry (Kharchoufi et al. 2018). Furthermore, numerous studies have demonstrated that pomegranate peel exhibits antibacterial and anti‐inflammatory properties, prompting researchers to utilise this product in cosmetics (Thilagavathi et al. 2016). The food industry is also very interested in this product; some food products, including cereals, yoghurt and ice cream, contain added pomegranate peels. The antioxidant and antimicrobial properties of pomegranate peel extend the shelf life of some meat products by 2–3 weeks in the refrigerator, making it a useful natural additive (Liu et al. 2023). The antioxidant activity of pomegranate peel is based on the reduction of reactive species, chelation of metal ions, strengthening of antioxidant enzymatic activity, regulation of the nuclear factor (erythroid‐derived 2)‐like 2 (Nrf2) signalling pathway and reduction of lipid peroxidation (Zhuang et al. 2019). The properties and uses listed for pomegranate peel are valid as long as the chemical composition remains stable; alteration of individual compounds could affect the content of the molecules and, consequently, their functions (Diao et al. 2022).

### 3.1 Extraction Techniques for Bioactive Compounds of Pomegranate Peel

The use of pomegranate peel in the food sector, as a natural antioxidant, involves the extraction of its bioactive components. An “extraction technique” is a method of separating a component from a mixture, exploiting property differences such as solubility, volatility or affinity for a component (Smaoui et al. 2019). The extraction techniques of the innumerable polyphenols contained in the pomegranate peel are divided into two broad categories: on the one hand, there are the traditional methods that use considerable volumes of extraction solvents and various manual processes, which make the results non‐standardizable. On the other hand, green extraction methods have been developed to overcome the limitations set by traditional methods. The latter also provides for higher extractive yields, smaller equipment and fewer processing steps (El Maaiden et al. 2022). The traditional extraction method for recovering phenolic components involves immersing the pomegranate peel in solvents such as water, methanol, chloroform, acetone, ethanol and ethyl acetate (Alara et al. 2021; Rahnemoon et al. 2018). The results showed that water has a better capacity for the extraction of total phenolic content, which is higher than that obtained with other solvents (Pathak et al. 2017). In parallel, another study highlighted that the best solvent for separating components with antioxidant activity from pomegranate peel was ethanol 70% (Magangana et al. 2021). Extraction efficiency increases proportionally with temperature, probably because higher values increase solubility, reduce viscosity and facilitate the passage of solvents through the solid substrate. However, the temperature should not exceed 40°C, as many chemical structures could alter and reduce or lose functionality (Rafi et al. 2021). Another method of traditional extraction is Soxhlet extraction, in which the material is subjected to various solvents, including water, ether, hexane, chloroform, benzene, methanol, acetonitrile and ethanol, which capture the desired molecules. The Soxhlet machinery separates the various components according to their polarity and that of the solvent. The least polar compounds of the dry material are extracted using the less polar solvents, like petroleum ether. Conversely, high polarity compounds are extracted with highly polar solvents (Sharmin et al. 2016). The limits of this type of extraction are the time required, the consumption of large amounts of solvent and the energy expenditure (Hasan et al. 2018). Among the green extraction techniques, there is one that uses pressurised liquid solvents (pressures > 4 MPa) and medium‐high temperature (through HPLC). High pressure facilitates the ability of solvents to penetrate the pores of the matter and the dissolution and separation of the components (Pereira et al. 2021). Another type of Ultrasound Assisted Extraction is based on the phenomenon of acoustic cavitation, which involves the creation of bubbles and their subsequent breaking, causing the release of bioactive substances (Souza et al. 2021). Extraction can also be assisted by enzymes; the principle involves the hydrolysis of food materials with an enzyme that acts as a catalyst to release bioactive components such as polyphenols and other phytochemicals. The most commonly used enzymes are pectinases, proteases and cellulases (Cano‐Lamadrid et al. 2022). When pomegranate peel is not subjected to extraction methods and is discarded or treated as waste, it would be advisable to recover it to exploit its countless benefits.

### 3.2 Recovery of Pomegranate Peel

This paragraph describes the main points to be made, should the pomegranate peel be recovered. According to the Food and Agriculture Organisation (FAO), the food processing and production stages are responsible for approximately 33% of the final product loss, and foods such as fruits, and vegetables can lose up to 50% (Priya et al. 2023). The pomegranate peel makes up, as previously mentioned, 50% of the total fruit weight, meaning there are roughly 3.6 million tons of peel generated annually worldwide. This huge amount of waste not only causes environmental pollution but also results in the loss of bioactive compounds that are beneficial for human health (Campos et al. 2022). It would be ideal to recover these beneficial compounds from the pomegranate peel, but it is also important to optimise the manufacturing process to preserve its phytochemical, biological and physical properties. First, the characteristics of the starting material can vary due to factors such as the growing region, fruit cultivar, climate and cultivation practices (Mirdehghan and Rahemi 2007). The pomegranate peel is characterised by a high degree of humidity, which is responsible for rapid alteration; therefore, it is necessary to treat it adequately to allow for its subsequent use. A dehydration process is advisable to reduce the moisture content of the pomegranate peel and prevent degradation, reduce enzymatic activity, limit fermentations and prolong its shelf life (Onwude et al. 2016; Sagar and Kumar 2010). The drying method is influenced by various factors such as the type of product, drying conditions, the availability of the drying equipment, the cost of operation and drying efficiency (Pateiro et al. 2022). The second step of processing the pomegranate peel is the extraction method and the solvent used (Benedetti et al. 2023). In compliance with this, three parameters are fundamental: the time necessary to carry out the drying technique, the temperature maintained and the solvent used for extraction (Kiran et al. 2024). The best conditions of these variables were those that determined a better yield of polyphenols, flavonoids, or vitamins content (Belwal et al. 2022). Numerous conventional methods of drying have been assessed in the literature, such as sun drying, hot air drying, freeze‐drying, microwave drying and fluidised bed drying.

The longest times occur when the drying method involves direct exposure to the sun: from 3 to 5 days, depending on the thickness and consistency of the product to be dried. The leathery consistency of pomegranate peel requires 5 days of exposure to the sun (Akpinar 2010). The positive consideration is that this method is inexpensive, while the downside is that it requires a lot of space, time and offers few hygienic and microbiological controls. Hot air drying also takes a long time (3–4 days for pomegranate peel); this method is simple, effective and relatively inexpensive (Kumar et al. 2017). The limitation of this technique is that it can completely dehydrate the surface of the product, forming cracks or a heterogeneous result (Polat 2024).

The drying ovens require a use of approximately 48 h for pomegranate peel and are equipped with a ventilation system to eliminate humidity from the material in a controlled way. This process avoids the formation of pests and reduces costs (Tekgül and Erten 2022).

Cold drying or freeze drying takes advantage of low temperatures and a treatment time of approximately 16–24 h. It is a process of removing water to preserve perishable materials by freezing them, then sublimating the ice into steam under vacuum conditions. This technique removes water without altering the structure, extends shelf life and reduces the weight of the product. In addition, freeze drying preserves the nutritional and organoleptic qualities, maintaining the freshness and quality of the dehydrated products. The pomegranate peels require 20 h (El‐Said et al. 2014).

Microwave drying is a very fast technique that takes up to 4–9 min. In fact, microwaves pass through and excite the water molecules present in the material, generating internal heat that makes it evaporate faster and more uniformly. Therefore, this technique reduces drying time, improving energy efficiency. These microwaves cause polar molecules, such as water, to rotate or vibrate, creating heat through friction between them. This heat causes the molecules of the substance to move more rapidly, uniformly and gradually absorb water (Kumar et al. 2017). Microwave drying of pomegranate peels has several positive points: speed, energy saving, cost reduction, uniformity of the result and maintenance of the high quality of the final product. A negative point of this method could be caused by an uneven distribution of power or airflow.

Often, some of these methods are used together to optimise moisture removal. The time required for methods of drying of pomegranate peel is associated with the temperature used: exposure to the sun requires a very high temperature (≅100°C); drying with hot air requires a temperature between 70°C and 200°C; drying in ovens requires a temperature between 50°C and 70°C; drying with microwaves requires temperatures of 35°C–50°C; freeze drying approximately −70°C (El‐Said et al. 2014).

**FIGURE 2:**
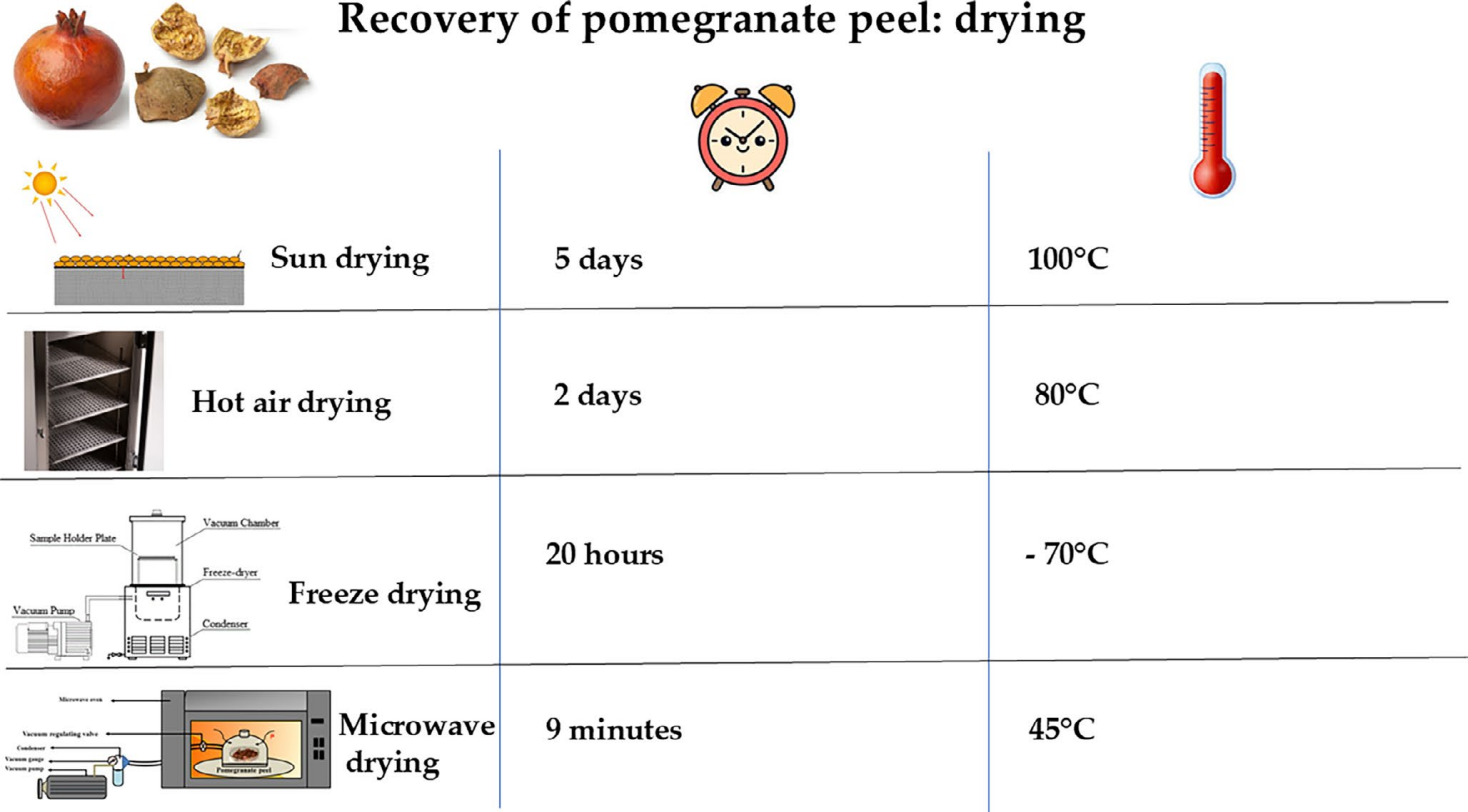
The drying process during pomegranate peel recovery is illustrated.

Finally, the last variable to consider is the choice of solvent for the extraction between water, methanol, ethanol, acetone and ethyl acetate (John et al. 2017; Kremer et al. 2023). In addition to water extraction, as already mentioned, methanolic extracts from pomegranate peel have also been shown to provide very positive results on the yield of bioactive compounds (Feng et al. 2022; Singh et al. 2002). A summary map of the pomegranate peel recovery and drying process is represented in Figure 2.

## 4 Peel of *P. granatum* and Neurodegeneration

Developed countries have seen significant increases in life expectancy in recent decades, as well as a rise in age‐related neurodegenerative diseases. Sadly, effective therapies for most of these conditions are still lacking; we only have symptom relief. As noted, consuming fruits and vegetables provides numerous bioactive compounds essential for maintaining physical and mental well‐being, thereby reducing the risk of neurodegeneration (Wang et al. 2011). Neuroinflammation, a nervous system inflammatory response primarily driven by microglia, is often linked to this phenomenon. Neurodegeneration and neuroinflammation are interrelated processes that play an important role in nervous system diseases, and neuroinflammation may be a contributing factor to the progression of neurodegeneration and neuronal deterioration (Mehdi et al. 2022). Brain inflammation disrupts neuronal communication and causes neuronal death (Maiuolo et al. 2018). Studies have shown that *P. granatum* has improved brain neurochemistry, inhibiting NF‐kB—known for its importance in regulating the immune and inflammatory responses—decreasing COX2, involved in the management of pain and inflammation, enzymatic activity (Yu et al. 2020) and caspase enzyme catalytic activity (essential in implementing apoptotic cell death (Sun et al. 2013)). Pomegranate peel has been shown to improve neuronal development, synaptic plasticity, learning and cognition, while also reducing lipid peroxidation and the levels of inflammatory cytokines (Velagapudi et al. 2015). Furthermore, it has been found to inhibit acetylcholinesterase, a key enzyme involved in certain neurodegenerative diseases (Gates et al. 2022).

### 4.1 Mechanisms Involved in Protection by Pomegranate Peel Against Neurodegeneration

**FIGURE 3:**
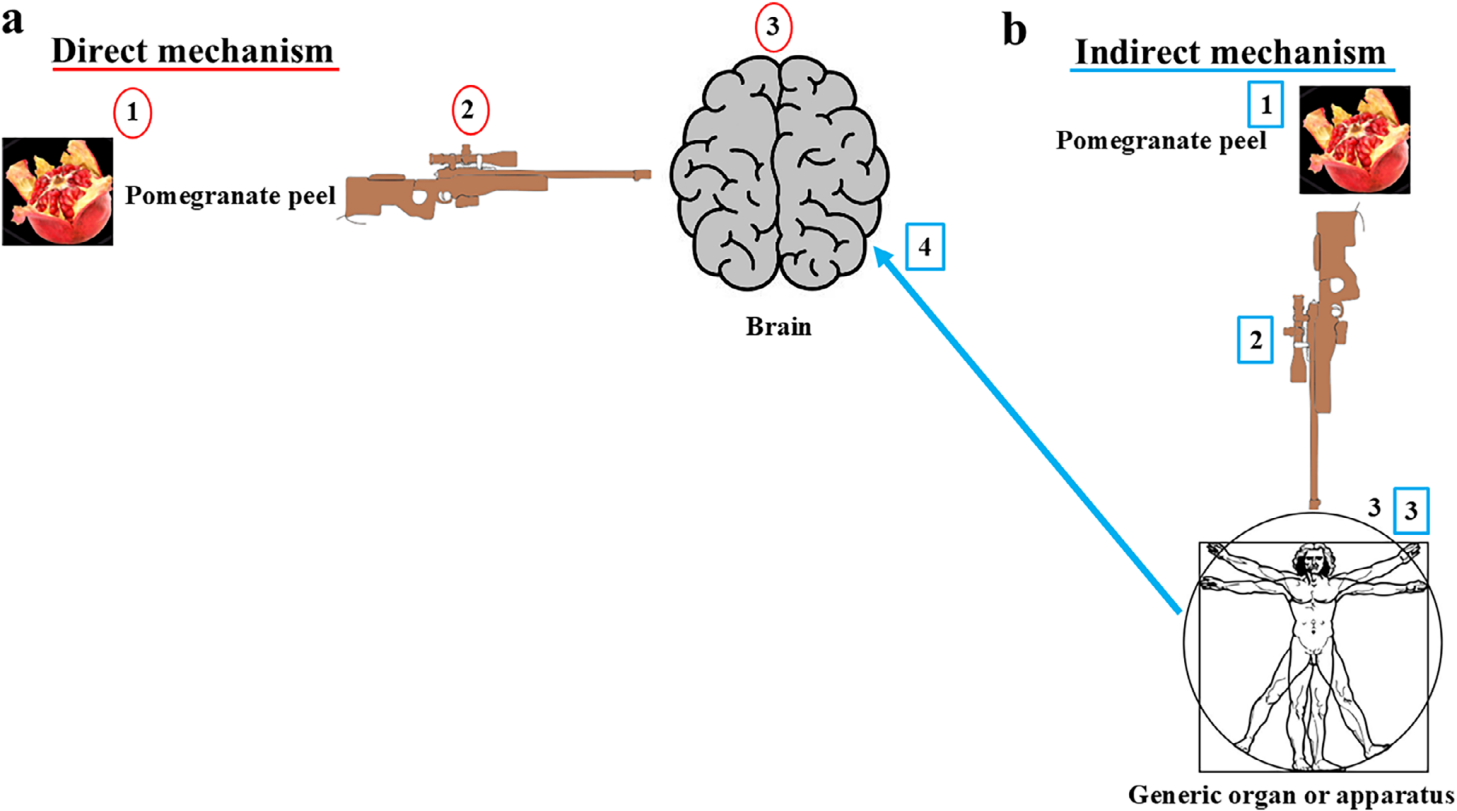
Direct and indirect mechanisms. In panel (a), the direct mechanism is shown. The continuation of events is highlighted by the numerical sequence indicated in red. (1) Treatment with pomegranate peel is carried out, and (2) can reach directly to the brain (3), acting beneficially against neurodegeneration. Panel (b) shows the indirect mechanism. The continuation of events is highlighted by the numerical sequence indicated in blue. (1) Treatment with pomegranate peel is carried out, and (2) can reach any organ or apparatus (3). The effect of the latter causes benefits on the brain against neurodegeneration.

The protection of pomegranate peel against neurodegeneration uses mechanisms of action that can be defined as ‘direct’ and ‘indirect’. In the direct mechanism, an active component of the fruit portion in question acts on the brain through a direct connection. Conversely, the indirect mechanism involves the effect of pomegranate peel on another organ or apparatus, which subsequently acts on the brain. This concept is graphically represented in Figure 3.

#### 4.1.1 Direct Mechanisms

Many neurodegenerative pathologies are linked to a persistent inflammatory state (Kalkan et al. 2024; Tsuji and Molnár 2025; Irwin and Vitiello 2019). The anti‐inflammatory effect, exerted by the pomegranate peel, has been found to produce beneficial effects on neurodegenerative diseases (Kwon and Koh 2020; Aleksandrova et al. 2023). Alzheimer's disease is characterised by the accumulation of unfolded proteins (beta‐amyloid) and neurofibrillary tangles (tau proteins) in the brain, both of which are responsible for toxic deposits and the subsequent death of neurons (Venusova et al. 2021). Experimental data reported that treatment with pomegranate peel, rich in ellagic acid and punicalagin, in an animal model of mice affected by Alzheimer's disease, resulted in a significant reduction in beta‐amyloid plaques and neurofibrillary tangles in the brain (Mphahlele et al. 2016). An in vivo study reported that mice in an Alzheimer's disease model, treated with pomegranate peel, showed positive results on the Barnes‐Wallis labyrinth test, with improved spatial memory in the search for the escape pathway. Furthermore, these mice showed a notable reduction in the number of plaques compared to untreated mice. This interesting result can be attributed to ellagic acid and punicalagin, which inhibited beta‐amyloid plaque formation (Urganci et al. 2025; Ciccone et al. 2023). In another study, conducted with the same animal models, it was highlighted that treatment with pomegranate peel reduced the levels of malonyldialdehyde and increased catalase and glutathione levels, compared to the counterpart with AD (Kwak et al. 2005). Finally, it was shown that the effects of pomegranate peel were more potent than the single molecules ellagic acid or punicalagin. The higher effect of peel extract compared to single components could be attributed to the copious amount of bioactive molecules contained in the peel, which probably act simultaneously through multiple pathways (Kwak et al. 2005). The observed protective effects could be explained by the anti‐inflammatory property exerted by punicalagin. Indeed, data available in the literature suggest that this compound can reduce the levels of beta‐amyloid_1–42_, TNF‐α, IL‐6, IL‐1β and the neuronal expression of the enzyme Beta‐Secretase 1 (BACE1), a key aspartate protease in the brain, critical for the production of beta‐amyloid peptide (Ahmed et al. 2014). The in vivo results were also confirmed in vitro; the treatment of human neuroblastoma cells with punicalagin highlighted the reduction in the expression of cyclooxygenase 2 (COX 2), responsible for the biosynthesis of molecules involved in inflammation (Kim et al. 2017). Furthermore, the treatment with punicalagin (10, 20 and 50 μM) in a co‐culture model, composed of astrocytes and a liver cell line, highlighted the reduction of COX 2 and BACE1 gene expression (Ahmed et al. 2014). In addition to reducing inflammation, the antioxidant mechanism, with the elimination of free radicals, can also contribute to the observed neuroprotection (Kim et al. 2017).

Amyotrophic lateral sclerosis (ALS) is a neurodegenerative disease characterised by loss of motor neurons, leading to progressive body weight loss, muscle weakness and paralysis (Harakeh et al. 2020). Congenital dysfunctions in ALS include mitochondrial alteration, redox imbalance, glutamate excitotoxicity, autophagic dysregulation, changes in RNA metabolism and astrocytic and microglial activation (Brown and Al‐Chalabi 2017). The accumulation of damaged mitochondria, an increase in oxidative stress and a reduction in ATP levels are responsible for cellular damage and apoptosis (Al‐Chalabi et al. 2014). In ALS, the functional alteration of the mitochondria contributes to the activation of astrocytes and microglia, which trigger a massive inflammatory response (Lou et al. 2020). To determine whether mitophagy played a central role in ALS, mice with the disease were treated with urolithin A (50 mg/kg/day), an activator of mitophagy. The results demonstrated that this compound improved motor function, mitigated neuroinflammation, alleviated muscle atrophy and fibrosis and reduced motor neuron loss. The strategy of urolithin A has been the activation of autophagy and mitophagy (Ziff et al. 2022).

Parkinson's disease (PD), a progressive neurodegenerative disease, is caused by degeneration of neurons that produce dopamine, a neurotransmitter essential for movement control. PD pathogenesis is characterised by the accumulation of Lewy body deposits (mainly alpha‐synuclein and ubiquitin) in the midbrain dopaminergic neurons (Zhang et al. 2025). It manifests with motor symptoms, such as tremor, slowness in movements, muscle stiffness and postural instability, but also with non‐motor disorders, including anxiety, depression, alterations in mood, sleep problems and cognitive decline (Schneider and Alcalay 2017). The creation of an animal model that mimics PD is carried out with an acute and high‐dose treatment of manganese chloride (MnCl_2_) in the brain (Nair et al. 2018). MnCl_2_‐induced neurotoxicity in rats occurs through several mechanisms, including oxidative stress, perturbation of neurotransmitter production and metabolism, neuroinflammation, endoplasmic reticulum stress and apoptosis (Nadig et al. 2022). In particular, dysregulation of glutamate, norepinephrine, serotonin and dopamine occurs, resulting in motor dysfunction (Pajarillo et al. 2022). Treatment with the polyphenol punicalagin (30 mg/kg, orally using stomach gavage) showed a significant improvement in locomotion, recovery of norepinephrine, serotonin and dopamine neurotransmitters, a decrease in the process of degeneration of the cell nucleus, maintenance of the correct histological structure and preservation of brain tissue (Nyarko‐Danquah et al. 2020). Furthermore, punicalagin has been shown to overcome the MnCl_2_‐induced damage, reducing both oxidative stress, inflammatory cascades and apoptosis (Kang et al. 2019; Yan et al. 2016). However, these effects could be explained predominantly by the antioxidant activity of the polyphenols contained in pomegranate peel. In fact, rats with PD, fed standardised pomegranate extract (40% ellagic acid) or exposed to punicalagin, showed attenuation of oxidative damage, reduction of malondialdehyde, reduction of inducible nitric oxide synthase (iNOS) activity, improved neuronal survival, locomotion and postural stability (Ali et al. 2022).

Multiple sclerosis (MS) is a chronic autoimmune disease affecting the brain and spinal cord, caused by a progressive demyelination responsible for interruptions in the transmission of nerve signals, axonal damage and neurodegeneration. MS is characterised by visual, sensory and motor disorders along with fatigue, muscle spasms, bladder and bowel dysfunction, paralysis and cognitive impairment (Subkorn et al. 2021). The animal model of Experimental Autoimmune Encephalomyelitis (EAE) is widely used to study multiple sclerosis. Under these experimental conditions, mice showed development of neuroinflammation, demyelination and axonal loss (Dendrou et al. 2015). The administration of ellagic acid (50 mg/kg) significantly alleviated the symptoms observed in the animal model, protecting the brain from damage, reducing pro‐oxidant and inflammatory effects, reducing demyelination, axonal loss and neuroinflammation, attenuating microgliosis and preventing apoptosis of oligodendrocytes (Robinson et al. 2014). Ellagic acid has protective effects on multiple sclerosis models, but it is not decisive. Ellagic acid could act both by exploiting its anti‐inflammatory potential, reducing IL‐17 (a pro‐inflammatory protein crucial for the immune system that, if in excess, is involved in autoimmune diseases) and antioxidant activity, attenuating cerebral lipid peroxidation and inhibiting demyelination (Depaz et al. 2011; Binyamin et al. 2015). However, it is desirable that its long‐term use may be associated with neuronal protection and fewer adverse effects compared to the synthetic drugs currently used for this disease (Kiasalari et al. 2021).

Huntington's disease (HD) is an inherited and progressive neurodegenerative disease that affects the central nervous system. It is characterised by the repetition of trinucleotide (cytosine‐adenine‐guanine) in the huntingtin gene (Htt), leading to the production of an abnormal protein, called huntingtin, which damages neurons in specific areas of the brain (Volpi et al. 2019). Characteristic clinical features are not only involuntary movement, cognitive and mental disorders, but also skeletal muscle atrophy, unintended weight loss and gastrointestinal dysfunction (Cepeda and Tong 2018). Since mitochondrial abnormalities and redox imbalance are major pathogenic factors in Huntington's disease, using antioxidants could attenuate disease progression. Ellagic acid (50 and 100 mg/kg) showed improvements in the cognitive abilities of rats affected by the disease (Wasser et al. 2020). Another compound that demonstrated excellent potential in the treatment of HD was urolithin A, which, by improving mitochondrial function, could slow the progression of the disease (Sharma et al. 2021).

#### 4.1.2 Indirect Mechanisms

**FIGURE 4:**
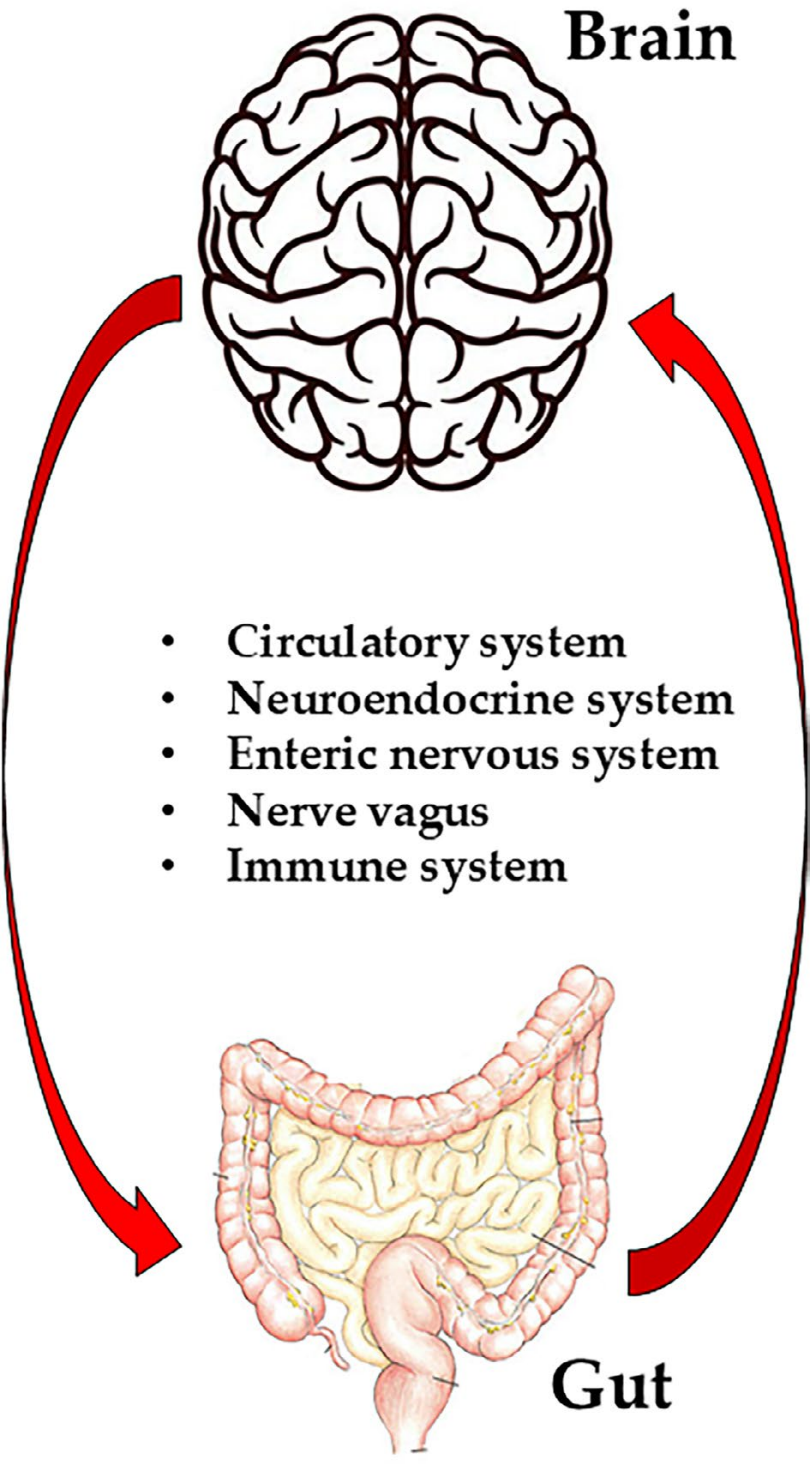
A close connection between the brain and the intestine. The systems that ensure the bi‐univocal connection between the brain and the intestine are shown.

The compounds contained in the pomegranate peel also act on the brain through the intestine. The intestine is responsible for much more than digesting the food ingested: it contains millions of cells and neuronal fibres which constitute a real nervous system operating in complete autonomy and which, independently of the central nervous system, can give rise to specific actions, such as intestinal contractions or release of digestive enzymes (Darwish et al. 2023). The intestine can also integrate and process the stimuli that the body receives, both internal and external and interact with the central nervous system. The intestine affects the brain and its development, while mental stress and emotions can irritate the intestine, causing inflammation and gastrointestinal disorders (Barbero Mazzucca et al. 2024). Intestinal inflammation can lead to neurological problems such as anxiety, depression and cognitive difficulties, emphasising the importance of a healthy gut for overall well‐being (Barrio et al. 2022). Changing one district causes the other to malfunction (Liu et al. 2022). Conditions of strong emotional stress can activate the circuits of anxiety and fear. The latter causes an increase in intestinal motility, resulting in the release of cytokines. In this way, the intestinal mucosa becomes irritated and inflamed and, in the most serious cases, can give rise to pathologies or even serious conditions such as irritable bowel syndrome or inflammatory bowel disease (Ferreiro et al. 2023). The intestine and the brain influence each other and it is precisely in this way that they influence, positively or negatively, the individual's state of physical and emotional well‐being. The gastrointestinal tract and the central nervous system can interact via the gut‐brain axis, which ensures two‐way communication through (1) the modulation of the immune system, (2) the involvement of the vagus nerve, (3) the enteric nervous system (SNE), (4) the neuroendocrine system and (5) the circulatory system (with production of neuroactive substances, such as metabolites and hormones) (Wang and Wang 2016). Figure 4 summarises the mechanisms that ensure gut‐brain interaction.

Another mechanism that correlates the intestine with the brain is the gut microbiota. The body's microbiota comprises all microorganisms (bacteria, fungi, viruses and protozoa) inhabiting areas in contact with the external environment—skin, mouth, nose, ears, eyes, lungs, gut and vagina—without causing harm (Chitnis and Weiner 2017). The intestinal microbiota is the most extensive, accounting for about 70% of the total (Di Vincenzo et al. 2023). In the human gut, only a few bacterial divisions have been identified: *Bacteroidetes*, *Proteobacteria*, *Firmicutes*, *Actinobacteria*, *Verrucomicrobia* and *Fusobacteria* (Adak and Khan 2019). Each individual is composed of different bacterial species, so the composition of the intestinal microbiota can be considered unique, much like a fingerprint (Pant et al. 2023). A healthy gut microbiota is characterised by adequate biodiversity and a good balance between beneficial and disadvantageous microorganisms (a condition known as eubiosis) (Álvarez‐Mercado and Plaza‐Diaz 2022). When the balance is altered (dysbiosis), a pathological condition is established that affects not only the digestive system but also the whole organism, generating neurological, cardiovascular, oncological and psychiatric dysfunctions (Sánchez et al. 2017).

Preclinical and clinical studies confirmed that microbial intestinal dysbiosis is responsible for the pathogenesis of central nervous system disorders, including Alzheimer's disease, Parkinson's disease, Huntington's disease, amyotrophic lateral sclerosis, multiple sclerosis, prion disease and frontotemporal lobar degeneration (Manos 2022), but also neuropsychiatric disorders such as autism spectrum, epilepsy and major depressive disorder (Kowalski and Mulak 2019). In particular, gastrointestinal symptoms often precede neurological symptoms several years before the development of neurodegenerative diseases (Block and Hong 2005). Changes in gut bacteria increase gut permeability and immune activation, causing widespread inflammation (Spielman et al. 2018).

The intestine and the entire bacterial population, gut microbiota, are considered key players in the health‐disease binomial and are often referred to as the ‘second brain’ (Seo et al. 2023). The main neurodegenerative diseases that affect the brain and that show, at the same time, an alteration of the intestinal microbiota are Alzheimer's disease, Parkinson's disease, multiple sclerosis, autism spectrum disorder, epilepsy and major depressive disorder (Ghasemi et al. 2021).

Recent studies have suggested that intestinal dysbiosis and *Staphylococcus*, *Streptococcus*, *Citrobacter*, *Salmonella*, *Klebsiella*, *Mycobacteria* and *Bacillus* genera can trigger protein misfolding, accumulate Aβ structures in the brain, and exacerbate neuroinflammation (Ray et al. 2025). Another study was conducted in vivo on mice in which Alzheimer's disease was transgenically induced; these animals had Aβ plaques, neurofibrillary tangles, reactive gliosis in the brain, memory defects, loss of integrity of the intestinal barrier, intestinal inflammation and alteration of the microbiota. These dysfunctions have been improved following the transplantation of microbiota from healthy wild‐type mice. Mice showed decreased levels of Aβ and tau proteins, improved synaptic plasticity, and a composition of intestinal microbiota similar to wild‐type mice (Catanzaro et al. 2015).

Several studies have shown a strong interconnection between intestinal dysbiosis and ALS; in fact, ALS is linked not only to alterations in the composition of the intestinal microbiota but also to the production of intestinal neurotoxins (tetanus and botulinum toxins) (Jyothi et al. 2015). These toxins could cross the intestinal lumen and reach the systemic circulation due to increased endothelial permeability (Wilson et al. 2010). Recent studies have reported that patients with ALS have low levels of anti‐inflammatory bacteria (*bacteroidetes*) compared to pro‐inflammatory bacteria (*Cyanobacteria*) (Uesaka et al. 2016).

PD patients reported prodromal nonmotor symptoms 20 years before the onset of the disease, including dysphagia, constipation, nausea, hypersalivation and altered bowel habit (Stenson et al. 2024). Studies conducted on animal models have shown that PD occurs in the enteric nervous system and spreads from the intestine to the brain via the vagus nerve (Gotkine et al. 2020). Some experimental data support a close connection between PD and gut microbiota: when mice affected by PD were treated with antibiotics and deprived of the intestinal microbiota, the symptoms of the pathology improved significantly; moreover, when germ‐free mice received a faecal microbial transplantation from PD patient donors, the animals showed an increased neuroinflammatory state and motor dysfunction (Wu et al. 2015).

Intestinal dysbiosis is one of the main factors responsible for MS (Li, Meng, et al. 2023), and patients reported this disorder. MS patients exhibited a reduction in anti‐inflammatory bacteria and an increase in pro‐inflammatory bacteria, resulting in a microbial intestinal profile that differed significantly from that of healthy individuals. Additionally, germ‐free mice exhibited higher myelin expression than sick mice (Pellegrini et al. 2020).

A correlation between intestinal dysbiosis and HD is usually observed before the onset of disease, when altered circulating intestinal metabolites (secreted by the enteric nervous system) are measurable (Dugger et al. 2017). In addition, there is evidence of an increase in the level of *Bacteroidetes* and a decrease in the level of *Firmicutes*. The *Firmicutes*/*Bacteroidetes* (F/B) ratio is a biomarker of the intestinal microbiota and can provide information on the body's ability to metabolise nutrients. A high ratio, with more *Firmicutes*, has been associated with obesity, while a lower ratio, with more *Bacteroidetes*, has been linked to a lean constitution. This specific intestinal dysbiosis may indicate a reduction in body weight (typical of the disease) despite increased dietary intake (Zhou et al. 2022).

**FIGURE 5:**
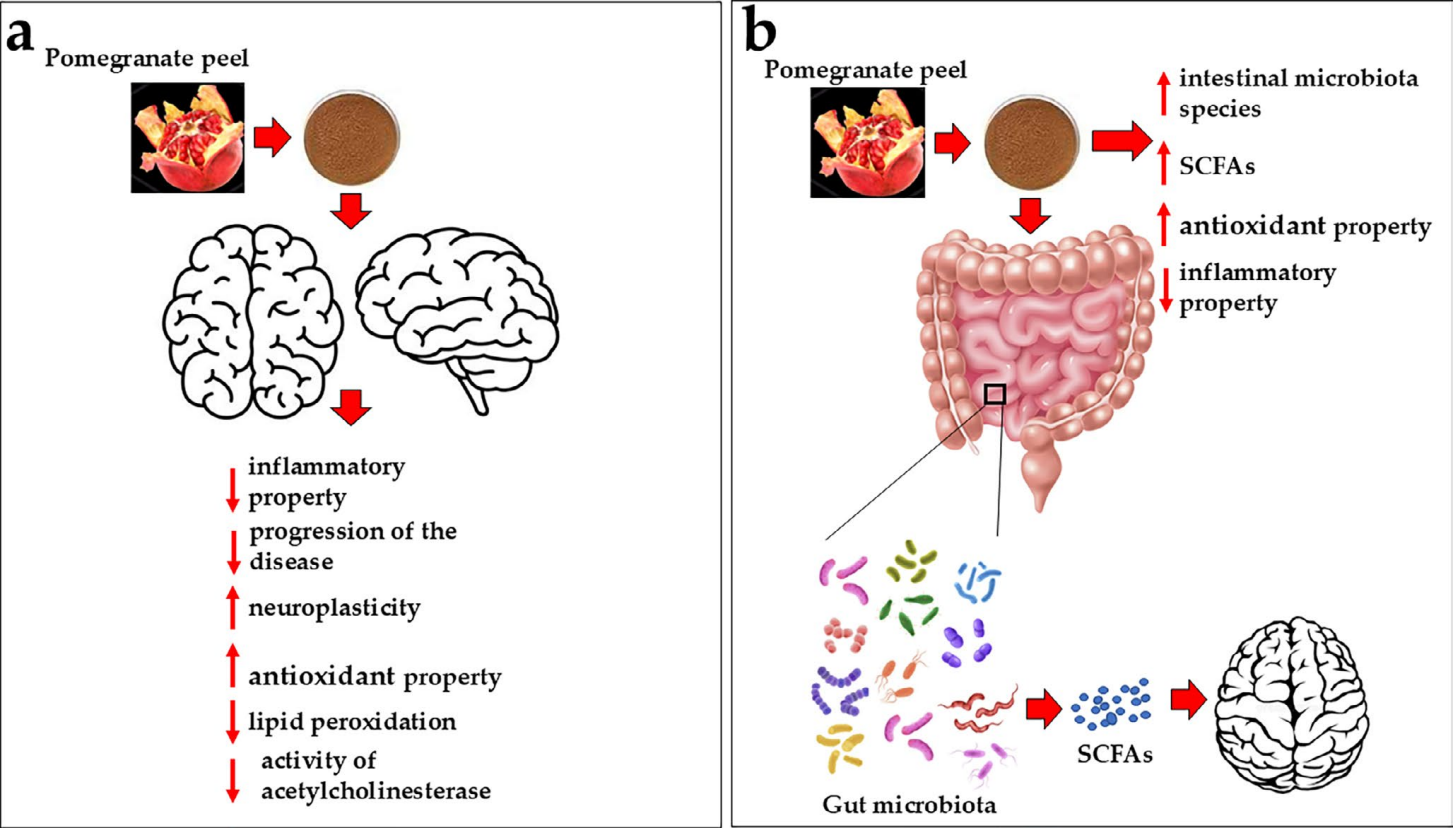
Protective effects of pomegranate peel on neurodegeneration. In panel (a), it is shown that the pomegranate peel can act directly on the brain, decreasing inflammatory properties, disease progression, lipid peroxidation and acetylcholinesterase activity; in addition, it can increase neuroplasticity, antioxidant properties and protective activities. In panel (b), the intestine takes on the pomegranate peel and, as a result, increases the variability of the species that make up the intestinal microbiota and their products, short‐chain fatty acids (SCFAs). Microbiota bacteria and SCAFs act beneficially on the brain.

Recent studies have shown that the consumption of pomegranate peel can enrich the intestinal microbiota: in particular, the polyphenols contained in the peel increase the abundance of specific bacterial metabolites (Mowry and Glenn 2018). Research on human gut microbiota revealed that pomegranate peel treatment boosts gamma‐aminobutyric acid (GABA), a fundamental inhibitory neurotransmitter in the human central nervous system. Its main function is to reduce neuronal arousal, helping to regulate anxiety, stress and sleep. GABA neurotransmitter production may potentially ease Parkinson's motor symptoms (Gubert et al. 2022). Moreover, studies indicate that pomegranate peel stimulates the production of short‐chain fatty acids (SCFAs) (van der Burg et al. 2011); intestinal bacteria ferment dietary fibre and resistant starch, generating SCFAs such as butyrate, acetate and propionate. SCAFAs protect intestinal health by fortifying the intestinal barrier, influencing motility and promoting epithelial cell growth. In addition, SCFAs may also affect the brain via the gut‐brain axis (Sorrenti et al. 2019). Pomegranate peel polyphenols boost beneficial gut bacteria like *Bacteroides*, *Lactobacilli*, *Christensenellaceae* and *Ruminococcaceae* (Mosele et al. 2015). Figure 5 summarises how the functioning of the brain is linked to the intestine.

## 5 Discussion

Neurodegeneration involves the deterioration and loss of neurons, which is responsible for the onset of neurodegenerative diseases and alterations in cognitive and/or motor functions. These events result from various neuroinflammatory processes involving alterations in proper glia–neuron communication. Although the causes that determine the onset of a neurodegenerative disease may be different, there is always a common biochemical pathway that characterises them and explains the loss of neuronal cells (George et al. 2023). In a non‐pathological context, astrocytes are multifunctional cells that ensure brain homeostasis, regulating blood flow (Fiacco and McCarthy 2006) and extracellular pH (Boyarsky et al. 1993), maintaining synaptic transmission (Ventura and Harris 1999) and carrying out the removal of free radical species of the brain (Drukarch et al. 1998). Microglial cells are dynamic immunocompetent cells, termed ‘brain‐resident macrophages’. Their role is to phagocytose neuronal debris, maintain brain homeostasis and ensure central nervous system activities, including neuronal survival (Yang et al. 2004), brain development (Bilimoria and Stevens 2015), synaptic remodelling and neurogenesis (Shin et al. 2020). Conversely, under adverse conditions, microglial cells become chronically activated and adopt the pro‐inflammatory M1 phenotype. The latter involves the expression of numerous inflammatory cytokines such as IL‐1β, IL‐6, interferon alpha (INF‐α), tumour necrosis factor alpha (TNF‐α) and chemokines. When the M1 phenotype persists, it leads to chronic neuroinflammation (Ismail et al. 2020), oxidative stress (Cheignon et al. 2018), blood–brain barrier disruption (Galea 2021; Huang et al. 2021), reduced neuronal viability, neuronal apoptosis (Hu et al. 1998), brain atrophy and astrocyte hyper‐reactivity (Danesi and Ferguson 2017). Pomegranate peel phytochemicals and polyphenols may play an important role, reducing the risk of developing both chronic inflammation and oxidative stress‐related diseases (Alami et al. 2024). Ellagic acid, ellagitannins, punicalagin and urolithins are just some of the beneficial components found in pomegranate and particularly in the fruit peel. Yet their role in protecting against neurodegeneration has become evident, as they are capable of extinguishing the oxidative potential and inflammatory processes involved in neurodegenerative diseases (Chang et al. 2019). It has been shown that the effects of pomegranate extracts or fruit peel are greater than those of the individual components. When natural products simultaneously contain many phytochemicals (flavonoids, anthocyanidins, anthocyanins, phenolic acids, hydrolyzable tannins, carotenoids) and this characteristic accentuates some protective effects, we can identify them as “superfoods” (Zhu et al. 2015). These phenomena also include the pomegranate fruit, which, by containing excellent categories of polyphenols, presumably amplifies their effects. Globally, polyphenols can interact with each other or with other plant components; this combined interaction produces a total synergistic effect, which is greater than the sum of the effects of individual molecules (Gigliobianco et al. 2020).

It is important not to forget that pomegranate peel is considered a waste product, even if its components can have a protective activity against neurodegenerative diseases, acting directly on the brain or, indirectly, on the same organ, but starting from the intestine (Hrelia et al. 2023). Therefore, it would be optimal to recover these valuable, sustainable and easily accessible resources from waste, thanks to a circular economy process (Hrelia et al. 2025; Jalila et al. 2016). The circular economy is an economic model that minimises the extraction of new resources, keeps materials and products in use for as long as possible and promotes reuse and recycling (Ojha et al. 2020). This approach not only reduces environmental impact but can also lead to new economic and trade opportunities (Osorio et al. 2021). Pomegranate peel can be considered a highly sustainable portion of the fruit and a confirmation of the success of the circular economy in the efficient recovery of agri‐food waste (Ballistreri et al. 2024). Another key point to discuss is that pomegranate peel improves the gut microbiota, regulating gut bacteria, increasing positive bacteria and decreasing harmful ones (Chen et al. 2025; Jose et al. 2017). Prebiotics are non‐digestible substances that promote the growth of beneficial bacteria in the intestine, contributing to the balance of bacterial flora and well‐being. These compounds are fermented by intestinal bacteria, producing short‐chain fatty acids (SCFAs) that have positive effects on the intestine and general health (Noor et al. 2022). Pomegranate peel fully complies with this definition and, for this reason, we can state that this waste product is a prebiotic.

## 6 Conclusions and Perspectives

Although this review has highlighted some interesting potential for using pomegranate peel, some limitations have emerged that could be addressed in future perspectives: first of all, data from human clinical trials involving neurodegenerative diseases are lacking, and the available data are few and do not include significant numbers. Second, the available results do not address the actual ability of pomegranate peel components to reach the neuronal compartment, the interaction of polyphenols with the blood–brain barrier and their penetrability.

## Author Contributions

Conceptualization: J.M., V.M. (Vincenzo Mollace), C.M. Writing: J.M., V.M. (Valeria Mazza), R.C. Original draft preparation: F.O., S.N., S.R. Editing: S.I., L.C.P., L.T., G.T. All authors have read and agreed to the published version of the manuscript.

## Funding

This work was supported by: Progetto ECS00000024 Rome Technopole SAP01‐2023‐000077 ‐ CUP B83D21014170006 ‐ PNRR Missione 4 Componente 2 Investimento 1.5, finanziato dall’Unione europea – NextGenerationEU; PON‐MIUR 03PE000_78_1, and PON‐MIUR 03PE000_78_2; Italian Ministry of Health [Ricerca corrente]. Open access funding provided by Università degli Studi Magna Graecia di Catanzaro within the CRUI‐CARE Agreement.

## Conflicts of Interest

The authors declare no conflicts of interest.

## Data Availability

Data sharing not applicable to this article as no datasets were generated or analysed during the current study.
